# Submerged Technique of Partially De-Epithelialized Free Gingival Grafts for Gingival Phenotype Modification in the Maxillary Anterior Region: A Case Report of a 34-Year Follow-up

**DOI:** 10.3390/medicina59101832

**Published:** 2023-10-15

**Authors:** Won-Bae Park, Wonhee Park, Philip Kang, Hyun-Chang Lim, Ji-Young Han

**Affiliations:** 1 Department of Periodontology, School of Dentistry, Kyung Hee University, Seoul 02447, Republic of Korea; 2 Private Practice in Periodontics and Implant Dentistry, Seoul 02771, Republic of Korea; 3 Department of Prosthdontics, Division of Dentistry, College of Medicine, Hanyang University, 222-1 Wangsimni-ro, Seongdong-gu, Seoul 04763, Republic of Korea; 4 Division of Periodontics, Section of Oral, Diagnostic and Rehabilitation Sciences, College of Dental Medicine, Columbia University, New York, NY 10027, USA; 5 Department of Periodontology, Periodontal-Implant Clinical Research Institute, School of Dentistry, Kyunghee daero 23, Dongdaemoon-gu, Seoul 02447, Republic of Korea; 6 Department of Periodontology, Division of Dentistry, College of Medicine, Hanyang University, 222-1 Wangsimni-ro, Seongdong-gu, Seoul 04763, Republic of Korea

**Keywords:** de-epithelialization, free gingival graft, gingival phenotype, gingival recession, root coverage

## Abstract

A coronally advanced flap combined with a subepithelial connective tissue graft is considered the gold standard for achieving root coverage on exposed root surfaces. Nevertheless, challenges arise when this technique is applied to multiple teeth and when the palatal soft tissue is very thin. Several surgical modifications have been reported to simultaneously achieve both single or multiple root coverage and widening of the keratinized gingiva. In this context, there have been no reported cases utilizing the submerged technique with partially de-epithelialized free gingival grafts. We intend to introduce a submerged technique involving partially de-epithelialized free gingival grafts for the modification of soft tissue phenotypes in the maxillary anterior region.

## 1. Introduction

Free gingival grafts are the most efficient procedure to increase the width of keratinized tissue and have been performed to cover denuded root surfaces [1,2]. However, free gingival grafts are limited for use in root coverage procedures due to esthetic issues and the limiting of blood supply to the denuded root surface. Free gingival graft engraftments rely on plasmatic circulation from the periosteum of the recipient bed. For the survivability of grafts, no dead space between the graft and the recipient bed should be present, and immobilization of grafts is essential [1]. Several techniques use thick free gingival grafts to increase the thickness of the graft to take advantage of the vascular network in the lamina propria or chemically detoxifying the denuded root surface using citric acid [3,4,5].

For root coverage, subepithelial connective tissue grafts are recommended over free gingival grafts. Coronally positioned flaps using a subepithelial connective tissue graft are the most predictable approach for covering denuded root surfaces [6]. Nonetheless, limitations exist when the roots of multiple teeth need to be covered or when the thickness of the palatal soft tissue is very thin. Therefore, an alternative surgical technique that can cover multiple denuded roots even in the presence of thin palatal soft tissue is needed. De-epithelialization of free gingival grafts for root coverage was introduced by Zucchelli et al. [7]. They reported that the root coverage technique using de-epithelialized free gingival grafts was as effective for root coverage and clinical attachment gain as a technique using subepithelial connective tissue grafts [7]. However, de-epithelialization may leave partial epithelium, so several complications such as superficial epithelial bands, cul-de-sac fluid, or epithelial cysts were reported [8].

De-epithelialized free gingival grafts have been used to increase the width of keratinized tissue or cover denuded root surfaces [9,10,11]. These techniques typically leave an epithelial layer in the coronal portion of the free connective tissue graft. To the authors’ knowledge, no procedure has been reported for submerging partially de-epithelialized grafts sparsely on the epithelial side of free gingival grafts into flaps.

In the present study, we introduce a submerged technique for partially de-epithelialized free gingival grafts to increase keratinized gingiva and root coverage for multiple teeth in the maxillary anterior region in patients with very thin palatal soft tissue.

## 2. Case Presentation

The patient, a 33-year-old male smoker, visited the Department of Periodontology, Kyung Hee Medical Center for treatment of cervical erosion in the maxillary anterior region in June 1988. Cervical erosion was observed in the upper six anterior teeth. The phenotype of the gingiva was thin. The thickness of the palatal mucosa was very thin (less than 2.0 mm). The quantity of subepithelial connective tissue grafting was not sufficient to cover the upper six anterior teeth. The submerged technique of a partially de-epithelialized free gingival graft was chosen to cover the denuded root surfaces.

**Figure 1:**
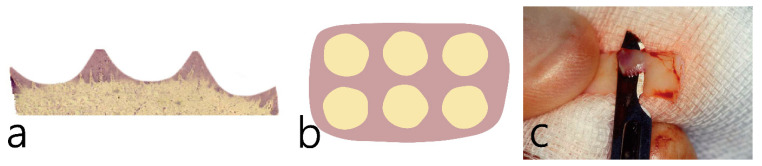
Schematic diagram after partial de-epithelialization of an about 1.0 mm thick free gingival graft obtained from the palate. (**a**) Partial de-epithelialization was performed. (**b**) Several portions of the epithelium remained. (**c**) Clinical picture of partially de-epithelialized free gingival graft using a #15 Bard-Parker blade.

A schematic diagram of a partially de-epithelialization with about 1.0 mm thick free gingival graft is shown in Figure 1a. The epithelium was partially removed and the lamina propria was exposed in several portions (Figure 1a). The obtained free gingival grafts were partially de-epithelialized using a #15 Bard-Parker blade (Figure 1b).

**Figure 2:**
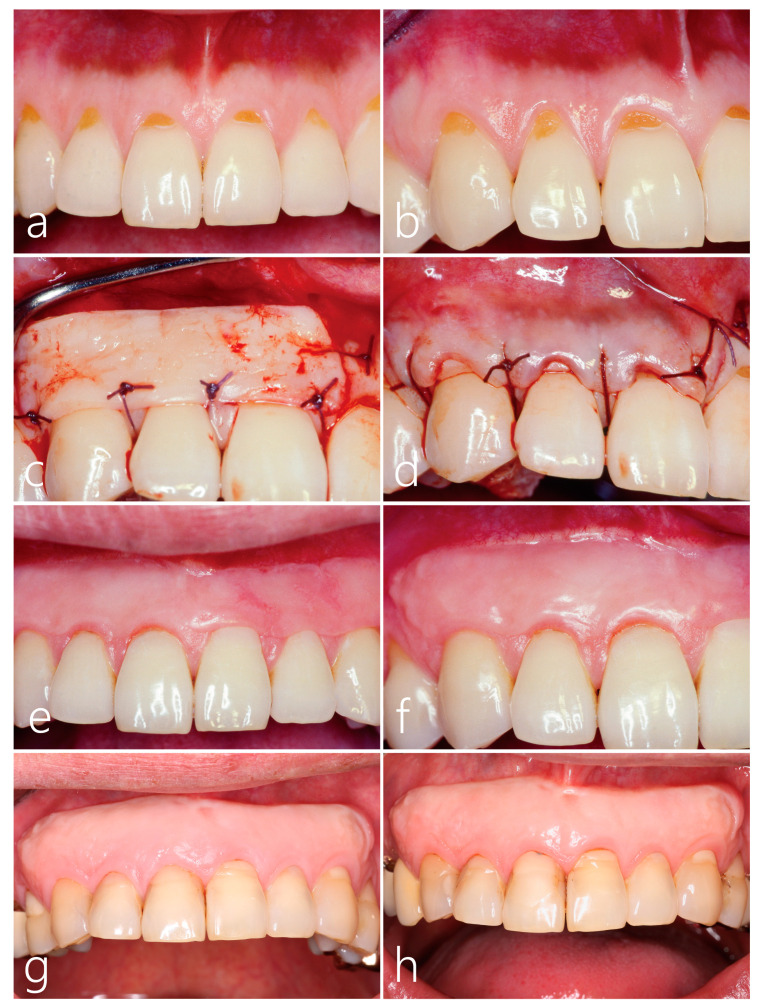
(**a**,**b**) Pre-operative clinical views of the maxillary anterior teeth with a thin biotype. Multiple cervical erosions were observed in the maxillary anterior teeth. (**c**) After the reflection of a split-thickness flap, a partially de-epithelialized graft was placed on the exposed root surface and stabilized with interrupted sutures. (**d**) The overlying flap was closed with 5-0 Vicryl^®^ suture material. (**e**) Two months after grafting, the width of keratinized gingiva was increased. (**f**) At two months after surgery, the overlying flap was sloughed away and the color of the graft appeared. (**g**) A clinical review at the 24-year follow-up shows well-maintained keratinized gingiva. (**h**) At the 34-year follow-up, there was minimal change in the width of keratinized gingiva with increased gingival thickness. However, a color discrepancy was observed.

All cervical portions of the maxillary anterior teeth were eroded and soft textured (Figure 2a,b). Therefore, the eroded surfaces were partially removed, and composite resin fillings were completed before the operation.

### Surgical Procedure

After local anesthesia with 2% lidocaine (containing 1:100,000 epinephrine), a split-thickness flap with two vertical incisions was reflected. Horizontal incisions were created to pass through the line angle between the cemento-enamel junction and the interdental papilla. In addition, de-epithelialization of the interdental papilla was performed to coronally position the flap. The excess resin fillings were slightly smoothed using an ultra-fine finishing bur and root planing procedures were performed. Root surface biomodification was not performed.

A free gingival graft was harvested from the ipsilateral palate. In the present case, the thickness of the palatal soft tissue was less than 2.0 mm. The thickness of a donor graft harvested through the conventional method of a free gingival graft procedure was approximately 1.0 mm. The donor site was sutured with 5-0 black silk for hemostasis and covered with a periodontal pack (Coe Pak^TM^, GC Inc., Alsip, IL, USA).

The partially de-epithelialized free gingival graft of about 1.0 mm in thickness was stabilized with 5-0 Vicryl^®^ (Ethicon Inc., Cincinnati, OH, USA) suture material (Figure 2c). The de-epithelialized part was directed toward the overlying flap rather than the root surface. After confirming the stabilization of the graft, the overlying flap was slightly coronally positioned and sutured with 5-0 Vicryl suture (Figure 2d). After two weeks, the same procedure was performed on the left side. Antibiotics and anti-inflammatory drugs were prescribed for 7 days. The patient was instructed to rinse their mouth with 0.12% chlorhexidine solution (Hexamedine, Bukwang Pharmaceutical, Seoul, Republic of Korea) for 2 weeks. Healing was uneventful and the sutures were removed after 2 weeks. Postoperative check-ups were conducted at 2, 4, 8, and 12 weeks. Thereafter, the patient was followed up once or twice per year. During the follow-up period, any dislodged resin restorations were refilled.

## 3. Results

Two months after surgery, complete root coverage and increased width of keratinized tissue was observed in the maxillary six anterior teeth (Figure 2e,f). Thin overlying split-thickness flaps were sloughed away and the original color of the free gingival graft was shown. Therefore, a color discrepancy was observed. At the 24-year follow-up, the width of keratinized tissue was well maintained (Figure 2g). Additionally, minimal changes in the gingival margins were exhibited. Complete phenotype modification of the periodontal soft tissue was observed. Thirty-four years after surgery, there were no additional changes of the periodontal soft tissue phenotype. Slight discolorations of the resin fillings were observed (Figure 2h).

**Figure 3:**
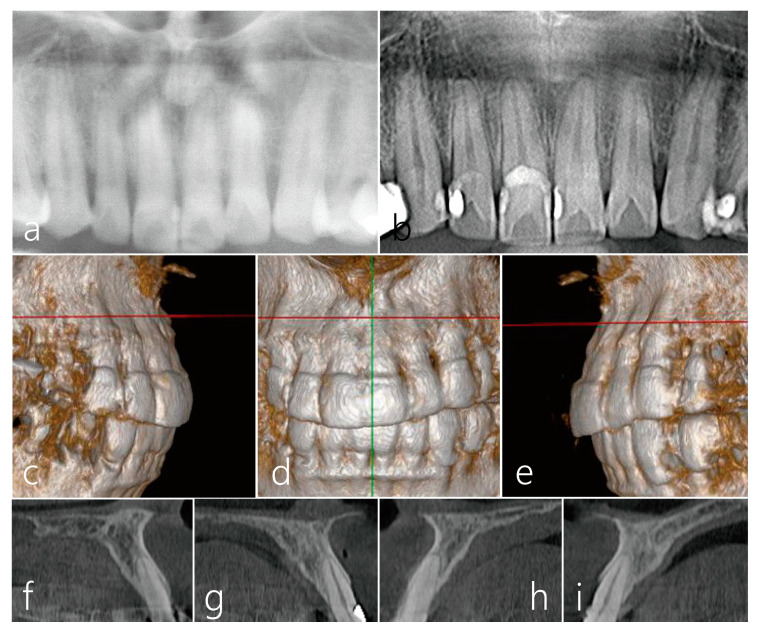
(**a**) A close-up image of panoramic radiograph at the 10-year follow-up. (**b**) Minimal radiographic bone change was observed in the maxillary anterior teeth on the close-up image of panoramic radiograph obtained 34 years after the procedure. (**c**–**e**) The 3-dimensional CBCT images taken 34 years after the procedure revealed well-maintained labial bone plates of the maxillary anterior teeth. (**f**–**i**) On the cross-sectional images of the CBCT at the 34-year follow-up, the labial bone plates of the maxillary anterior teeth were well-maintained.

The panoramic radiograph at 10 years after surgery revealed slight interdental bone loss in the maxillary anterior teeth (Figure 3a). Minimal changes in the interdental bone levels of the maxillary anterior teeth were observed in the panoramic radiograph during the 34-year follow-up after the procedure (Figure 3b). The three-dimensional CBCT images from the 34-year follow-up after the surgery revealed well-maintained labial bone plates of the maxillary six anterior teeth (Figure 3c–e). The cross-sectional images of the CBCT also show well-maintained labial bone plates of the maxillary anterior teeth (Figure 3f–i).

## 4. Discussion

The thin gingival phenotype has a high risk of long-term esthetic complications and is also associated with crestal bone loss [12]. As in the present case with multiple eroded roots and a thin gingival phenotype, the possibility of tearing or perforation of a split-thickness flap increases during reflection of the flap. In addition, if the thickness of the palatal donor site is very thin, donor site complications such as necrosis, dehiscence, and delayed wound healing may occur [7]. In this situation, modified techniques such as the collection of subepithelial connective tissue by de-epithelialization or partly epithelialized free gingival grafting have been reported as alternatives to the trap-door technique [7,13]. However, in cases where the thickness of palatal mucosa is very thin after the removal of epithelium, the remaining thickness of the de-epithelialized graft is not thick enough to contain lamina propria, which does not guarantee a successful result. In the present case, a partially de-epithelialized free gingival graft was devised for greater preservation of the lamina propria, and the submerged technique was established by inserting the graft under the flap for more vascularization. At 34 years after surgery, it exhibited very favorable results in terms of covering the denuded root and increasing the width of keratinized tissue except for the color discrepancy.

Several surgical procedures including a laterally sliding flap, a coronally advanced flap, and its combinations with barrier membranes or subepithelial connective tissue grafts have been proposed to treat gingival recession of a single tooth [14]. In cases where gingival recessions of multiple teeth exist, the selection of an effective procedure and the skill of the operator are important for successful outcomes. The coronally advanced flap with a subepithelial connective tissue graft is the most popular technique for the treatment of gingival recessions of multiple teeth [11,15,16]. To reduce patient discomfort, various types of allografts or replacement biomaterials have been used under a coronally advanced flap instead of a connective tissue graft [14,16]. In addition, technical modifications such as the pouch and tunnel techniques have been reported to increase the efficiency of the procedure [17,18,19].

Nonetheless, the coronally advanced flap with a subepithelial connective tissue graft has exhibited the most optimal clinical outcome [14,16]. However, if the thickness of the palatal mucosa is too thin to harvest adequate subepithelial connective tissue, another option is to use a free gingival graft. The use of a free gingival graft in root coverage has several limitations due to limited blood supply and color discrepancy. Therefore, several modifications of free gingival grafts, such as de-epithelization of all harvested grafts [6,7] or coronally advanced flaps after free gingival grafting [20], have been introduced.

Conventionally, the subepithelial connective tissue graft was harvested using a trap-door approach, a scalpel with parallel blades [21], or a single incision [22,23]. In the root coverage procedure, several authors have reported on the de-epithelialization of the free gingival graft instead of harvesting the subepithelial connective tissue graft [7,8,24,25]. Bertl et al. (2015) reported that the average palatal mucosal thickness in an autopsy study ranged from 2.35 to 6.89 mm, indicating that individual variation was very large [26]. They stated that the average thickness of the epithelium was 0.3 mm and the lamina propria thickness ranged from 0.2 to 3.3 mm. This indicates that there may be many cases where the trap-door approach is difficult due to the relatively shallow palatal mucosal thickness. Moreover, the connective tissue graft obtained from the de-epithelialized free gingival graft has sufficient lamina propria and is composed of relatively less glandular and fatty tissue compared to those obtained from the conventional trap-door approach [26,27]. In addition, a de-epithelialized free gingival graft exhibited less postoperative shrinkage and a greater increase in buccal gingival tissue thickness 1 year after root coverage compared to a conventional connective tissue graft [7]. This may be due to differences in the proportions of fatty/glandular tissue, which would influence shrinkage and compressibility by the overlying flap [26]. In addition, they reported that increased palatal mucosal thickness was mainly due to increased thickness of the submucosa since increased palatal mucosal thickness was not associated with increased thickness of the lamina propria.

In the conventional free gingival graft procedure, the mucosal flap is apically positioned or removed to prepare the recipient bed. However, in the present procedure using a partially de-epithelialized free gingival graft, a thin mucosal flap is placed over the partially de-epithelialized free gingival graft without being removed. In the early healing stages after surgery, the mucosal flap can provide blood supply to the underlying partially de-epithelialized free gingival graft. The thickness of the partially de-epithelialized free gingival graft is approximately 0.5 mm to 1 mm, consisting of epithelium and several portions of the lamina propria. It is theorized that the presence of the overlying flap, which allows for plasmatic circulation and additional vascularization, could have supported the retention of the thin graft. It is hypothesized that the double blood supply may contribute to the viability of a partially de-epithelialized free gingival graft on the exposed root surface. Furthermore, the overlying mucosal flap could serve as protection for a partially de-epithelialized free gingival graft against external stimuli during the initial stage of the healing process.

In the present procedure, it is important to ensure that the de-epithelialized aspect does not come into contact with the root surface, but instead remains in contact with the overlying mucosal flap. Contact between the outer epithelial surface and the root surface could hinder tissue attachment, potentially leading to the development of pockets or epithelial cysts [8,28,29]. In cases where de-epithelialization over the entire surface of the graft is performed, the retention of the overlying flap could lead to the formation of gingival pockets due to incomplete sloughing of the overlying flap [8]. However, in this case, since only a partial de-epithelialization was performed, the overlying flap was completely sloughed away and the formation of gingival pockets did not occur. The remaining epithelium in contact with the mucosal flap may have sloughed off, resulting in gradual loss of the thin overlying flap. Furthermore, it allowed for the preservation of the lamina propria to a greater extent when compared to the conventional de-epithelialized free gingival graft technique. The expression of color and texture of free gingival graft is the most challenging aspect [30].

It is reported that approximately 31–45% of the graft width tends to shrink within the first 3 months post-surgery, with minimal subsequent alterations thereafter [31]. Nevertheless, in the current case, minimal reduction in the width of the graft was observed 34 years after surgery. This discrepancy could be attributed to variations in the utilization of an overlying flap, since the partially de-epithelialized graft exhibited improved initial vascularization and plasma circulation in comparison to the conventional free gingival graft. There are limited studies on changes in graft thickness after free gingival graft procedures. Park et al. (2017) reported that the graft thickness around the implant increased 15 years after the free gingival graft procedure [32]. While the present case involved grafting on a natural tooth rather than an implant, it appears that the graft thickness has exhibited a significant increase over the course of the 34 years.

We report a rare case with a 34-year follow-up of a partially de-epithelialized free gingival graft performed on the multiple maxillary anterior teeth. This is an intermediate procedure between a free gingival graft and a subepithelial connective tissue graft. This procedure has the dual characteristics of a free gingival graft to increase the width of keratinized mucosa, and a subepithelial connective tissue graft for root coverage.

One disadvantage of this technique is that postoperative color mismatching is caused by the exposure of the epithelium of the underlying graft. However, the color mismatching of a partially de-epithelialized free gingival graft on multiple teeth is less obvious than that of a single tooth.

This case report has several limitations. First, clinical photos and panoramic radiographs were of poor quality as they were from 34 years prior. Second, several recordings for maintenance or clinical photos including donor site might have been missed, although most of the patient’s data and surgical procedures were well documented as a case note with clinical photos and radiographs. Third, the case is limited by the small number of cases and must be supported by further investigation into the effects of residual epithelium.

## 5. Conclusions

Within the limitations of the present case report, the submerged technique using partially de-epithelialized FGG exhibited an ability to increase the width of the keratinized tissue and enable effective root coverage on multiple teeth even when the thickness of the palatal mucosa is inadequate.

## Data Availability

Not applicable.

## References

[1] Miller P.D. (1987). Root coverage with the free gingival graft. Factors associated with incomplete coverage. J. Periodontol..

[2] Deo S.D., Shetty S.K., Kulloli A., Chavan A.K.R., Dholakia P., Ligade S., Dharmarajan G. (2019). Efficacy of free gingival graft in the treatment of Miller Class I and Class II localized gingival recessions: A systematic review. J. Indian Soc. Periodontol..

[3] Holbrook T., Ochsenbein C. (1983). Complete coverage of the denuded root surface with a one-stage gingival graft. Int. J. Periodontics Restor. Dent..

[4] Miller P.D. (1982). Root coverage using free soft tissue autografts following acid application. I. Technique. Int. J. Periodontics Restor. Dent..

[5] Jahnke P.V., Sandifer J.B., Gher M.E., Gray J.L., Richardson A.C. (1993). Thick free gingival and connective tissue autografts for root coverage. J. Periodontol..

[6] Chambrone L., Chambrone D., Pustiglioni F.E., Chambrone L.A., Lima L.A. (2008). Can subepithelial connective tissue grafts be considered the gold standard procedure in the treatment of Miller Class I and II recession-type defects?. J. Dent..

[7] Zucchelli G., Mele M., Stefanini M., Mazzotti C., Marzadori M., Montebugnoli L., de Sanctis M. (2010). Patient morbidity and root coverage outcome after subepithelial connective tissue and de-epithelialized grafts: A comparative randomized-controlled clinical trial. J. Clin. Periodontol..

[8] Ripoll S., de Velasco-Tarilonte Á.F., Bullón B., Ríos-Carrasco B., Fernández-Palacín A. (2021). Complications in the Use of Deepithelialized Free Gingival Graft vs. Connective Tissue Graft: A One-Year Randomized Clinical Trial. Int. J. Environ. Res. Public Health.

[9] Zucchelli G., Bentivogli V., Ganz S., Bellone P., Mazzotti C. (2016). The connective tissue graft wall technique to improve root coverage and clinical attachment levels in lingual gingival defects. Int. J. Esthet. Dent..

[10] Kinaia B.M., Kazerani S., Hsu Y.T., Neely A.L. (2019). Partly Deepithelialized Free Gingival Graft for Treatment of Lingual Recession. Clin. Adv. Periodontics.

[11] Zucchelli G., Marzadori M., Mounssif I., Mazzotti C., Stefanini M. (2014). Coronally advanced flap + connective tissue graft techniques for the treatment of deep gingival recession in the lower incisors. A controlled randomized clinical trial. J. Clin. Periodontol..

[12] Fawzy M., Hosny M., El-Nahass H. (2023). Evaluation of esthetic outcome of delayed implants with de-epithelialized free gingival graft in thin gingival phenotype with or without immediate temporization: A randomized clinical trial. Int. J. Implant Dent..

[13] Cortellini P., Tonetti M., Prato G.P. (2012). The partly epithelialized free gingival graft (pe-fgg) at lower incisors. A pilot study with implications for alignment of the mucogingival junction. J. Clin. Periodontol..

[14] Cairo F. (2017). Periodontal plastic surgery of gingival recessions at single and multiple teeth. Periodontol. 2000.

[15] Langer B., Langer L. (1985). Subepithelial connective tissue graft technique for root coverage. J. Periodontol..

[16] Chambrone L., Ortega M.A.S., Sukekava F., Rotundo R., Kalemaj Z., Buti J., Prato G.P.P. (2018). Root coverage procedures for treating localised and multiple recession-type defects. Cochrane Database Syst. Rev..

[17] Dembowska E., Drozdzik A. (2007). Subepithelial connective tissue graft in the treatment of multiple gingival recession. Oral Surg. Oral Med. Oral Pathol. Oral Radiol. Endo..

[18] Dani S., Dhage A., Gundannavar G. (2014). The pouch and tunnel technique for management of multiple gingival recession defects. J. Indian Soc. Periodontol..

[19] Lee Y., Lee D., Kim S., Ku Y., Rhyu I. (2021). Modified tunneling technique for root coverage of anterior mandible using minimal soft tissue harvesting and volume-stable collagen matrix: A retrospective study. J. Periodontal. Imp. Sci..

[20] Caffesse R.G., Guinard E.A. (1978). Treatment of Localized Gingival Recessions. Part II. Coronally Repositioned Flap with a Free Gingival Graft. J. Periodontol..

[21] Harris R.J. (1992). The connective tissue and partial thickness double pedicle graft: A predictable method of obtaining root coverage. J. Periodontol..

[22] Hurzeler M.B., Weng D.A. (1999). Single incision technique to harvest subepithelial connective tissue grafts from palate. Int. J. Periodontics Restor. Dent..

[23] Kumar A., Sood V., Masamatti S.S., Triveni M.G., Mehta D.S., Khatri M., Agarwal V. (2013). Modified single incision technique to harvest subepithelial connective tissue graft. J. Indian Soc. Periodontol..

[24] Bakhishov H., Isler S.C., Bozyel B., Yıldırım B., Tekindal M.A., Ozdemir B. (2021). De-epithelialized gingival graft versus subepithelial connective tissue graft in the treatment of multiple adjacent gingival recessions using the tunnel technique: 1-Year results of a randomized clinical trial. J. Clin. Periodontol..

[25] Bednarz W., Majer J., Pakuszy´nska-Błaszczyk J., Dominiak M., Gedrange T., Zielinska-Pałasz A. (2021). Coronally Advanced Flap in the Treatment of Multiple Adjacent Gingival Recessions along with a Connective Tissue Graft Harvested from Augmented or Nonaugmented Palatal Mucous Membrane: A Two-Year Comparative Clinical Evaluation. Appl. Sci..

[26] Bertl K., Pifl M., Hirtler L., Rendl B., Nürnberger S., Stavropoulos A., Ulm C. (2015). Relative Composition of Fibrous Connective and Fatty/Glandular Tissue in Connective Tissue Grafts Depends on the Harvesting Technique but not the Donor Site of the Hard Palate. J. Periodontol..

[27] Zuhr O., Bäumer D., Hürzeler M. (2014). The addition of soft tissue replacement grafts in plastic periodontal and implant surgery: Critical elements in design and execution. J. Clin. Periodontol..

[28] Harris R.J. (2002). Formation of a cyst-like area after a connective tissue graft for root coverage. J. Periodontol..

[29] de Castro L.A., Vêncio E.F., Mendonça E.F. (2007). Epithelial inclusion cyst after free gingival graft: A case report. Int. J. Periodontics Restor. Dent..

[30] Harris R.J. (1998). Treatment of a previously placed autogenous masticatory mucosa graft (free gingival graft). A case report. J. Periodontol..

[31] Silva C.O., Ribeiro E.D.P., Sallum A.W., Tatakis D.N. (2010). Free gingival grafts: Graft shrinkage and donor-site healing in smokers and non-smokers. J. Periodontol..

[32] Park W.B., Kang K.L., Han J.Y. (2017). Long-Term Clinical and Radiographic Observation of Periimplant Tissues after Autogenous Soft Tissue Grafts: A 15-Year Retrospective Study. Implant Dent..

